# White-Tailed Deer Spatial Distribution in Relation to ‘4-Poster’ Tick Control Devices in Suburbia

**DOI:** 10.3390/ijerph19084889

**Published:** 2022-04-17

**Authors:** Patrick Roden-Reynolds, Cody M. Kent, Andrew Y. Li, Jennifer M. Mullinax

**Affiliations:** 1 Department of Environmental Science and Technology, 1422 Animals Sciences Bldg., University of Maryland, College Park, MD 20742, USA; paddy16@umd.edu (P.R.-R.); cmkent@umd.edu (C.M.K.); 2 Invasive Insect Biocontrol and Behavior Laboratory, USDA-ARS, 10300 Baltimore Ave., Beltsville, MD 20705, USA; andrew.li@usda.gov

**Keywords:** 4-Poster, bait, deer, integrated pest management, movement, *Odocoileus virginianus*, suburban, supplemental feeding, ticks

## Abstract

Deer are keystone hosts for adult ticks and have enabled the spread of tick distributions. The ‘4-Poster’ deer bait station was developed by the United States Department of Agriculture to control ticks feeding on free-ranging deer. Although effective in certain scenarios, ‘4-Poster’ deer treatment stations require the use of bait to attract deer to one location, which may cause increased deer disease transmission rates and habitat damage. To better understand and manage the impact of baited ‘4-Poster’ stations on deer movements, we captured and GPS-monitored 35 deer as part of an integrated pest management project. Fifteen ‘4-Poster’ stations were deployed among three suburban county parks to control ticks. To quantify the effects of ‘4-Poster’ stations, we calculated deer movement metrics before and after feeders were filled with whole kernel corn, and we gathered information on visitation rates to feeders. Overall, 83.3% of collared deer visited a feeder and revisited approximately every 5 days. After feeders were refilled, collared deer were ~5% closer to feeders and conspecifics than before filling. Males used a higher percentage of available feeders and visited them more throughout the deployment periods. Although these nuanced alterations in behavior may not be strong enough to increase local deer abundance, in light of infectious diseases affecting deer populations and effective ‘4-Poster’ densities, the core range shifts and clustering after refilling bait may be a cause for concern. As such, trade-offs between conflicting management goals should be carefully considered when deploying ‘4-Poster’ stations.

## 1. Introduction

Deer are keystone hosts for adult ticks, and among other species, have been implicated in the overall rise in tick abundances and outbreaks of tickborne diseases in the past several decades in the United States [1,2,3,4,5,6]. Regardless of deer’s competence as a reservoir for specific pathogens, high deer densities support large tick populations and move ticks through the environment [5,6,7]. Past research has led to convoluted conclusions regarding various deer management strategies to reduce tick abundances in the hopes to control tick-borne disease prevalence or risk [2,8,9]. Researchers and managers have tried a wide range of strategies, such as deer removal [8,9,10], exclusion [11], and self-applied topical treatments as part of an integrated pest management (IPM) strategy [12,13,14].

One such IPM strategy is the ‘4-Poster’ feeder station (Dandux Outdoors, C.R. Daniels, Inc., Ellicott City, MD, USA) developed by the United States Department of Agriculture Agricultural Research Service (USDA-ARS) (Patent # 5,367,983) to attract deer to baited stations for the self-application of pesticides to control ticks [12,15]. While feeding on whole kernel corn from special bins, deer contact paint rollers saturated with specially formulated Tickicide^®^ (4-Poster Tickicide^®^, Y-Tex Corporation, Cody, WY, USA) with 10% permethrin as the active ingredient [16]. The literature pertaining to ‘4-Poster’ effectiveness for tick reduction is ample [13,14,17,18,19]. The ‘4-Poster’ feeders have been found to effectively reduce tick abundances in localized areas immediately surrounding the feeder or in an island situation but were deemed better suited for an integrated control approach utilizing multiple management tools [20]. However, use of bait (e.g., corn, mineral licks, etc.) as an attractant is a controversial management tool, and the aspect of feeding deer via the ‘4-Poster’ station has caused them to be illegal in several states.

In a review of the literature, we only found one study [21] that specifically investigated the effect of baited ‘4-Poster’ feeders on deer movements. However, that study did not activate ‘4-Poster’ feeders during summer months, though they are typically active from spring through fall to overlap with all peaks in tick life-cycle stage activity. We found no other papers exploring the number of visits of individual deer to ‘4-Poster’ feeders or the time between visits to ‘4-Poster’ feeders, and both metrics would have implications for any self-applied tick control program.

While the unintended effects of ‘4-Poster’ feeders on deer and deer behavior are less understood, there are many studies that have investigated the presence of supplemental feed or bait [22,23,24,25]. Supplemental feed or bait can be any artificial food source that is provided for the purposes of viewing, hunting, nutritional supplementation, or management [26,27]. However, feeding sites can concentrate animals in high densities and exacerbate ecological issues in localized areas such as browse damage [28], intraspecific competition that disproportionately restricts certain individuals from food sources [29,30], and disease transmission such as chronic wasting disease (CWD) [31,32,33]. Priesmeyer et al. (2012) reviewed past deer baiting papers and concluded that supplemental feeding can disrupt movement patterns, but impacts were highly variable. While baiting has been shown to concentrate animals in specific areas [32,34], it is not fully understood if baiting protocols used for ‘4-Poster’ operations increase site-specific deer densities on the landscape, especially in overpopulated suburban areas. Nor is it well understood how often or during which times deer visit ‘4-Poster’ feeders, which is potentially consequential for their overall utility.

The goal of our study was to quantify and evaluate the spatial distribution of white-tailed deer when ‘4-Poster’ feeders were fully integrated into an IPM program in a suburban county in Maryland. The specific research objectives of this study were to quantify the number of visits, time of visit, and duration of time between visits as well as to evaluate the spatial distribution of fine-scale occurrence distributions in relation to ‘4-Poster’ feeders by collared deer. We strived to further deer managers’ understanding of changes in deer behavior in relation to active ‘4-Poster’ feeders or other bait stations and enable the better implementation of ‘4-Poster’ feeders when their use is deemed appropriate.

## 2. Materials and Methods

### 2.1. Study Area

This research was conducted within 3 public parks in Howard County, Maryland, approximately 29 km south of Baltimore, MD, and 43 km north of Washington D.C. The study sites were within the metropolitan boundary of Howard County characterized by increased urban development and population density [35]. Within the metropolitan zone, there were 9.64 people/ha versus the more rural western portion of the county with 1.24 people/ha [35]. On average, annual rainfall was 1.09 m and annual snowfall was 0.58 m. In winter, the average temperature was 0.78 °C, and the average daily minimum temperature was −4.9 °C. In summer, the average temperature was 22.9 °C, and the average daily maximum temperature was 29.6 °C [35]. Forest cover within the study sites ranged from mixed hardwoods, predominantly oak (*Quercus* spp.) and Tulip poplar (*Liriodendron tulipifera*), to successional fields of black walnut and eastern red cedar. The understory was often dominated by invasives such as Autumn olive (*Elaeagnus umbellate*), Amur honeysuckle (*Lonicera maackii)*, and multiflora rose (*Rosa multiflora*). However, native species such as *Rubus spp*., spicebush (*Lindera benzoin)*, and greenbrier (*Smilax rotundifolia)* were common [35]. Parks differed in development and number of amenities, ranging from grassy trails to recreational fields, pavilions, and paved trails.

### 2.2. Trapping Methods

We captured deer using drop nets (15.2 m × 15.2 m) and box traps (0.9 m width × 1.22 m height × 1.83 m length) (Wildlife Capture Services, Flagstaff, AZ, USA) baited with whole kernel corn and apples from January to April in 2017 and 2018 [36]. We physically restrained and anesthetized deer via hand syringe in the gluteal muscle mass using BAM^™^ (Wildlife Pharmaceuticals, Windsor, CO, USA). The fixed-dose BAM^™^ formulation contained 27.3 mg of Butorphanol, 9.1 mg of Azaperone, and 10.9 mg of Medetomidine per 1 mL of solution. We administered BAM^™^ based on estimated weight according to label directions. After injection, we applied face blinds to deer and moved them onto a tarp for processing. During the processing period, we sexed each individual and estimated age by examining tooth wear and replacement [37]. We deployed Lotek GlobalStar L collars on individuals greater than 1 year old when they correctly fit. GPS collars remained on for a pre-programmed duration (~116 or 62 weeks, depending on deployment date) and recorded a GPS location and timestamp onboard every hour. GPS collars also attempted to remotely upload a subset of locations to a cloud service every third hour. When processing was complete, we reversed BAM^™^ with the intramuscular administration of Atipamezole (25 mg/mL) and Naltrexone (50 mg/mL) (Wildlife Pharmaceuticals, Windsor, CO, USA) in amounts based on the initial injection amounts of BAM™. We immediately released deer after recovery and monitored them until they exited the area. We used VHF to monitor collared deer every day for the first week of deployment and then once a month afterward.

### 2.3. ‘4-Poster’ Monitoring

We deployed fifteen ‘4-Poster’ feeders among 3 county parks to passively treat ticks on free-ranging deer in October 2017 (Table 1). We removed all feeders from the field at specific times each year to avoid interference with managed hunt operations, or they were allowed to remain empty during less active tick periods. We deployed ‘4-Poster’ feeders at rates of 1 feeder per 15–19 ha depending on the park. Specific feeder locations balanced perceived access for deer as well as ease of access for maintenance crews. Crew members visited each station every 1–3 weeks to refill corn supply, replenish Tickicide^®^ rollers, and perform general repairs.

### 2.4. Data Analysis

#### 2.4.1. Occurrence Distribution Analysis

We created 95% and 50% occurrence distributions (ODs) to delineate ranges of more precise representations of space used during specific, short time periods. These were created using Brownian Bridge density estimators and were based on where the animal was located during our specific observation periods [38]. To evaluate the interaction between ‘4-Poster’ feeders and ODs, we created two 3-day ODs, one before and one after each date of feeder service when it was restocked with corn. Feeders were serviced between 06:00 and 18:00 on the day of record. To avoid any impact of the hour of service, the pre-service OD ended at 06:00 on the day of feeder servicing, and the post-service OD began at 18:00 on the day of service.

During this study, we serviced each feeder 40–60 times depending on its location, but to increase the independence of pre- and post-service occurrence distributions, we only included feeder service dates that were ≥10 days since the previous servicing. Then, we only included feeder service dates when the feeder was found to be completely empty from the previous servicing to avoid skewing the analysis due to consistent corn availability. We used the resulting list of feeder service dates to create ODs for deer that had GPS data that overlapped with or fell within the pre- and post-service periods for each date. We assumed that feeders were empty (no corn available) during 3-day pre-service periods and full (corn available) during 3-day post-service periods.

Occurrence distributions were calibrated with 10 m error, derived from the average location error of field-tested collars, using ctmmweb [39,40]. Lastly, we only created ODs for deer that accrued at least 20 GPS points within each 3-day pre- and post-feeder service period. We analyzed each park as separate units because they exhibited variable numbers of feeders and different servicing schedules. We tested the effect of the feeders on deer behavior by comparing both the 95% and 50% OD size, OD Euclidean distance to all feeders, and OD Euclidean distance to the nearest feeder in the 3 days prior to feeder refill and 3 days after feeders were refilled using generalized linear mixed models (glmm) with the package *glmmTMB* [41]. For OD size, we log-transformed area to normalize it and used a Gaussian model, whereas we fit the distance to feeder models with a Tweedie distribution with a log link due to a large number of 0 s when the OD overlapped feeders [42]. Full models contained the fixed effects of before or after the feeder service date (treatment), park, sex, age as a categorical variable, and data upload type. All models also contained the random effects of individual deer IDs, feeder service week, and a factor variable identifying the paired before/after measurements. Both analyses of distance to feeder also contained a random effect of individual feeder ID. We conducted model selection by generating all potential subset models of the full model constrained to include all random effects and the effect of treatment using function *dredge* in the package *MuMIn* [43] and comparing with AICc [44]. We then tested the significance of the treatment effect with a Wald chi-square test.

#### 2.4.2. Proximity Analysis

To test for an impact on overall proximity to the nearest feeder in the hourly locations of deer, we fit a generalized additive model with the package *mgcv* [45]. We modeled distance to the nearest feeder, which was always positive, with a Gamma distribution and log link. The full model contained the fixed effects of treatment, park, age, sex, data upload type (full store-on-board dataset or remotely uploaded dataset), the smooth effect of hour, and smooth interactions between hour and sex as well as hour and age fit with a cyclic cubic regression spline. The model also contained random effects of individual deer IDs, the ID of the nearest feeder, and a factor variable identifying the paired before/after measurements, as well as a first-order autoregressive process (AR1). We removed the random effect of the feeder week prior to analysis as it had almost no effect and caused issues with convergence. Model selection and hypothesis testing were carried out again as stated above for the OD analysis.

#### 2.4.3. Cluster Analysis

To test if the presence of filled feeders caused deer to be more clustered, we generated two metrics, the mean nearest neighbor distance and average pair-wise distance between all collared deer at a park for each hourly interval for the 3 days before and after feeders were refilled. Again, we analyzed these as separate generalized additive models with Gamma distribution and log link. The full model contained the fixed effects of treatment, park, the smooth effect of hour molded as a cyclic cubic regression spline, the number of individuals with location data for that hour modeled as a cubic regression spline, and the random effect identifying the paired before/after measurements. The model also contained an AR1 process to control for correlated errors between consecutive hours. Model selection and hypothesis testing were carried out again as stated previously.

#### 2.4.4. Feeder Revisitation Analysis

To evaluate how deer visited feeders or the time until a feeder would be visited by a deer, we quantified the number of times movement paths crossed through a specified area of interest during active feeder deployment. The *recurse* package was used to conduct a revisitation analysis to ‘4-Poster’ feeder locations specified as areas of interest [46]. Using the *getRecursionsAtLocations* function, we specified ‘4-Poster’ feeder locations to gather metrics including the number of visits, entrance time, and time since the last visit. We chose a radius of 15 m to create locations of interest around ‘4-Poster’ feeders to account for an average GPS collar error of 9.6 m. Thus, a deer was detected visiting a feeder if its movement path intersected a 15 m radius circle around ‘4-Poster’ GPS locations. Revisitation analyses are sensitive to large gaps or irregularities in data, so data derived from remote uploads were not used for this analysis. Additionally, the time between feeder visits was log-transformed to normalize it, and variation was modeled with a generalized additive mixed model that included sex, park, a smooth effect of day of the year using a cubic regression spline, the interaction between the day of year and sex, and random effects of individual ID and feeder ID. We performed backward model selection with the constraints that we retained all random effects and variables in any interactions present, so all were lower-order effects.

Lastly, we modeled the probability of a specific deer visiting a feeder by converting visits identified by *recurse* into an hourly Bernoulli variable, where a visit was coded as 1 if *recurse* identified a revisit within that hour, and 0 if it was not. We included data between an individual deer’s first and last ‘4-Poster’ visit each year to ensure we only captured times when feeders and collars were operational, deer were in the study area, and feeders had been discovered. We modeled the probability of a deer visiting a feeder using a generalized additive mixed model as a binomial process with complementary log-log link because the probability of a visit in a given hour was very low [47]. The full model included fixed effects of sex and the smooth tensor product of hour and day, where an hour was fit with a cyclic cubic regression spline and a day with a regular cubic regression spline. The model also contained interactions between hour, day, and sex. We included deer ID as a random effect. We performed model selection as outlined previously. All statistical analyses were completed in program R [48]. Results were considered significant if *p* < 0.05.

## 3. Results

### 3.1. Deer Data

After selecting deer locations with timestamps that overlapped or fell within selected ‘4-Poster’ feeder pre- and post-service dates, we were able to create 36 unique ODs from eight (m = 4, f = 4) individual deer at Cedar Lane, 70 ODs from nine (m = 3, f = 6) individual deer at Blandair, and 64 ODs from eight (m = 2, f = 6) individual deer at Rockburn. The maximum number of locations per 3-day OD was 72, and the minimum was 20. For the revisitation analyses, we included deer that had GPS data overlapping with active feeder deployment periods. Eleven of the fifteen deer collared at Cedar Lane, ten of ten deer collared at Blandair, and nine of ten deer at Rockburn had overlapping data.

### 3.2. Effect of ‘4-Poster’ Feeders on Deer Spatial Distribution

**Figure 1:**
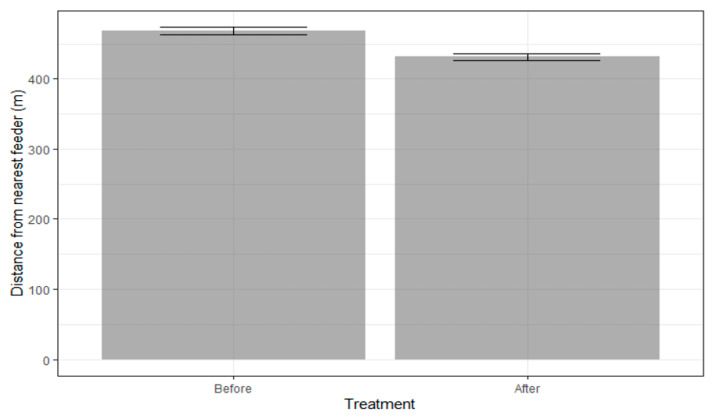
Average distance of hourly white-tailed deer locations to nearest feeder (+/−SE) both before and after feeder service dates in Howard County, Maryland 2017–2019.

Pre- and post-feeder servicing 95% occurrence distributions averaged 35.2 ± 44 and 31.9 ± 37.7 ha, respectively. The 50% OD of pre- and post-feeder servicing averaged 6.23 ± 9.3 ha and 5.6 ± 6.0 ha, respectively. Based on the high *p*-value of the ‘treatment’ effect, there was no significant difference in the size of the OD before or after feeders were filled (Table 2). In terms of distance to feeders, there was no significant difference in the distance from the 95% OD extent to all feeders nor to the nearest feeder (Table 3 and Table 4). Importantly, though, the 50% ODs were significantly closer to the nearest feeder after feeders were refilled (Table 4). Additionally, hourly GPS locations were significantly closer (4.8%) to feeders after they were refilled (Table 5; Figure 1). For conspecifics, the mean nearest neighbor between collared deer significantly decreased by 5.16%, and the average pair-wise distance between collared deer significantly decreased by 4.25% when feeders were refilled (Table 6).

### 3.3. Revisitation Analysis

Overall, 83.3% (*n* = 30) of collared deer were “detected” visiting a feeder. Eight of eleven deer at Cedar Lane, ten of ten deer at Blandair, and seven of nine deer at Rockburn visited a feeder. On average, deer at Cedar Lane visited one of two available feeders 17.5 times ± 39.3 (range: 0–134). Deer at Blandair visited two of four feeders an average of 24 times ± 32.2 (range: 1–104). Deer at Rockburn on average visited two of nine available feeder stations 31.4 ± 65 times (range: 0–201). Males (*n* = 12) visited 55.3% of available feeders at each park, whereas females (*n* = 18) visited 39.5% of available feeders. Males (revisits = 34.7 ± 61) also had a greater average number of revisits to feeders than females (revisits = 16.7 ± 32).

**Figure 2:**
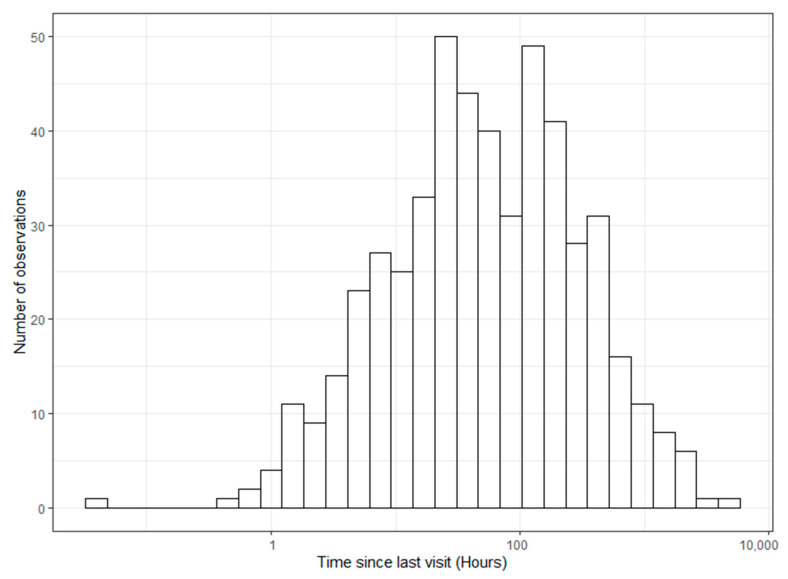
Histogram of log-transformed hours between visits to ‘4-Poster’ feeders by individual white-tailed deer in Howard County Maryland 2017–2019.

**Figure 3:**
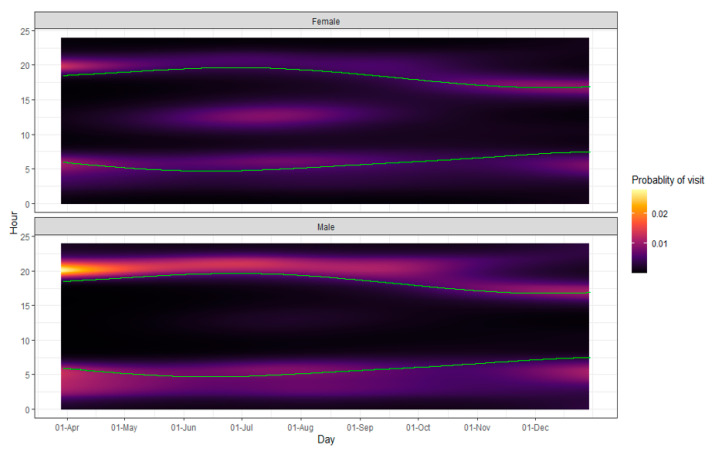
Model predictions for probability of visit to ‘4-Poster’ feeders by hour of day and day of year for female and male white-tailed deer in Howard County Maryland 2017–2019. Green lines indicate sunrise and sunset, and the probability of visit scale runs from ~0.009 to 0.03, an ~300% shift in magnitude.

When analyzing the store-on-board GPS collar datasets for the amount of time until a feeder would be visited by a deer, deer returned to feeder stations an average of every 5.04 days (*n* = 9; Figure 2), and the best model only contained random effects (deer ID and feeder ID) and the intercept. However, when modeling the probability of a deer visiting a feeder, the best-performing model contained all effects (Table 7). Feeder usage probabilities were expected to be low given it is the probability that a specific deer will visit a feeder during any given 1 h period throughout the entire year. In terms of major shifts in magnitude of the probability, interestingly, both sexes showed an increased probability of feeder usage during crepuscular hours, as well as mid-day during summer (Figure 3). However, males were more likely to use feeders during crepuscular hours and females during mid-day.

## 4. Discussion

We characterized white-tailed deer spatial distribution and visitation in relation to ‘4-Poster’ baited treatment stations to understand the influence of baited stations on deer movement and quantify deer use of tick treatment stations. Our study analyzed ‘4-Poster’ usage across a period of time and in ways that have not been studied in the past. Overall, we found evidence of small shifts in deer distributions over fine temporal scales in response to feeder stations being refilled with corn. We demonstrated small shifts towards feeding stations, alongside a complementary decrease in distance between collared deer. Presumably, those findings led to the small increase in the density of deer, or at least deer activity, we detected in the region around the ‘4-Poster’ feeders. In general, we found that collared deer visit feeders reasonably frequently throughout the year, with most individuals visiting more than one feeder within a park. Though both sexes primarily used feeders around crepuscular hours, we did document clear temporal differences in the probability of feeder visits. Interestingly, we detected changes in deer space use despite lacking sufficient samples from some age classes and using a 15 m proxy distance to feeders as a ‘visitation’. Overall, these findings give us a better understanding of both the effects of ‘4-Poster’ feeders on deer behavior and distribution, as well as on how deer use these feeders, pointing to clear management concerns and recommendations.

### 4.1. Impacts of Feeder Stations on Deer Distribution

Our findings on the impact of active ‘4-Poster’ feeders were generally consistent with past research. Though the literature reports that access to supplemental feed has had variable effects on white-tailed deer movements and spatial distribution, with studies generally reporting modest impacts, most have demonstrated that range size is likely to decrease in the presence of supplemental food [21,25,27,28,49]. We saw evidence that the distribution of hourly locations and proximity to conspecifics changed with the availability of corn in ‘4-Poster’ feeders on a fine temporal scale. Deer hourly locations were slightly closer to ‘4-Poster’ feeders and other collared deer during the 3 days following feeder refilling. However, this finding did not extend to the 95% OD shrinking after feeders were serviced, possibly due to decreased statistical power from the smaller sample size. However, our 50% OD was found closer to feeder stations following the refill. This finding was supported by past research which has shown that the distance from core areas to supplemental feed areas can shift 4.1–115 m closer once food is available [21,22,26], comparable to the average shift of ~35 m we documented. It is possible that corn was available at the feeders during a portion of the pre-servicing period, violating our assumption that feeders were empty. If corn was available during the pre-servicing period, this may cause deer to be closer to feeders, weakening our comparison between before and after periods. Similarly, the corn supply could have been completely depleted during the 3-day post-servicing period, but this is less likely as feeders were regularly stocked with 100–200 lbs of corn each service period.

Many studies have found that supplemental feed is unlikely to draw in more deer from outside locations, but if the feed is within an established range, then it can increase recruitment and compact ranges, leading to higher densities of deer [26,27]. Though our analysis did not look at recruitment, the documented shift in ranges towards feeders could lead to modestly elevated local densities. This is shown more directly in our analysis of deer clustering, where we documented collared deer being closer together after feeders were refilled, presumably in response to multiple deer shifting their ranges in the same direction. 

Overall, our results support local deer populations shifting their ranges towards filled feeders, concentrating deer at point sources of food and closer to conspecifics. Although these shifts are of fairly small magnitudes, clustering and increasing shared space among deer can have negative consequences to managing the spread of diseases such as CWD or bovine tuberculosis, which have important implications for humans [50,51]. It is impossible to know about feeder use by uncollared deer without monitoring stations with camera traps. While individual deer may partition times of feeder visits and avoid direct contact with other deer, disease agents such as the prions of CWD are environmentally persistent in feces, urine, and saliva, allowing deer to become passively infected. Informed decisions on the appropriate use of ‘4-Poster’ feeders in specific areas will depend on current knowledge of deer densities, tick densities, disease prevalence, and community needs and concerns. Regardless, managers must critically balance the conflicting outcomes of ‘4-Poster’ use, which include local tick control and the likelihood of concentrating local deer herds.

### 4.2. Use of ‘4-Poster’ Feeders by Deer

Some studies have suggested deploying ‘4-Poster’ feeders or supplemental feeding stations at one station per 50–60 ha to reduce deer using multiple bait piles while still effectively covering the area [21,26]. Based on recent literature reviews, ‘4-Poster’ densities may need to be much higher to reach the population size needed to achieve effective tick control [20]. Our densities were approximately one feeder per 15–19 ha, and we saw 83% of collared deer using feeders. We also documented the widespread use of multiple feeders by the same individual deer, and time between visits was highly variable among individuals and difficult to predict using sex or day of the year. Some individuals would consistently use specific feeders for months at a time, whereas others would visit only a few times in the same time period.

In terms of tick control, other studies have shown that the treatment of 50–70% of the deer population will result in a 60% reduction in infected nymphs after five years [52,53]. However, Tickicide^®^ is permethrin-based, which kills ticks on contact but does immediately degrade in the environment. Depending on exposure to light and precipitation, permethrin has a half-life averaging 39 days in the soil, but ranges from 1 to 113 days [54,55,56]. Given our visitation rates, to maintain consistent continuous treatment, deer may have to visit active feeders a minimum of once every 2 weeks in instances when new questing ticks are picked up after the last treatment of Tickcide^®^ has degraded naturally.

There are a number of factors that could influence deer use of feeding stations. Natural forage availability can influence feeder use [13,57]. Both sexes exhibited slightly lower probabilities of feeder visits during spring and fall compared to the rest of the year, possibly due to changes in food resource availability such as spring green-up or mast production in the fall. Additionally, personal preference and personality between individuals may have a large influence on which individuals use feeding stations and the frequency of use [25]. Overall, we found that males used a higher percentage of available feeders and visited them more throughout the deployment periods (Figure 3). Though we demonstrate that both sexes showed an increased probability in feeder use during crepuscular hours, Bartoskewitz et al. (2003) had also documented males exploited supplemental feed more heavily than females.

Social hierarchy has been shown to influence which deer have access to feeders [30,58]. Males and particularly older males will have dominance over resources, but they prefer to use supplemental feed after daylight hours, which may drive females to use feeders more often during the day. Consistent with this premise, we document a mid-day spike in feeder visits by females during the summer months (Figure 3). As such, it does not appear that social dominance by males alone prevents the use of ‘4-Poster’ feeders by females but may be one influence that shifts females’ use outside of the crepuscular hours, at least during summer. Unfortunately, we could not model the effect of age on feeder use due to the lack of sample size for certain ages, though age and dominance hierarchies can affect which deer have access to feed [29], and we hypothesized increasing age would increase dominance of use.

As days shortened, mid-day feeder use declined, and may have contributed to the decreased ability of females to access ‘4-Poster’ feeders. Unfortunately, the decrease in female mid-day visits September through October overlaps with peak activity season for adult black-legged ticks. Furthermore, rutting activity, which occurs primarily from 15 October to 15 November in this area, may disrupt feeder usage, as we see an increase in deer speed and activity coinciding with a lower probability of crepuscular ‘4-Poster’ visits during this time [59,60]. As a result, we recommend keeping ‘4-Poster’ feeders active until the end of December to allow more access for deer as the probability of use begins to increase again (Figure 3).

Many past studies only looked at baiting impacts for short periods within a single year such as hunting season or winter supplemental feeding. However, Jerina (2012) postulated that the length and history of supplemental feeding may be an important factor because it takes several years of continuous baiting to observe clear responses in home range size. Feeders for this study were first deployed in 2017, but there was a legacy of baiting and ‘4-Poster’ feeder use by County Park personnel for management activities in previous years, which may have made the local deer population more inclined to use supplemental feed. Deer use of ‘4-Poster’ stations may have been intensified because we were filling them on a timeline that allowed feeders to be emptied between feeder service dates. Thompson et al. (2008) found that deer use of feeders was more intense for rationed feed versus unlimited amounts, and our ‘4-Poster’ feeders were often found empty during most feeder service dates, creating a timeline of empty–full–empty bait availability. Based on past work and as 83.3% of our collared deer visited a feeder within a 3-year period and revisited feeders an average of every five days, we recognize our protocol may provide adequate coverage for tick control. When deemed necessary, we recommend keeping ‘4-Poster’ feeders active continuously throughout tick seasons for multiple years to achieve the best results in tick reductions. Unfortunately, in many regions, active tick seasons can occur year-round depending on climate and tick species present. Therefore, we recommend strategically restricting access at certain times (e.g., snow cover, air temp < 1.67 °C) to decrease costs, reduce pressure on immediate habitat, and intensify deer use of feeders once refilled.

## 5. Conclusions

Based on past studies and findings from this study, any similar ‘4-Poster’ or baiting protocol is likely to alter the spatial distribution and movements of white-tailed deer, resulting in slightly more compact space use and a shift in resident deer toward feed sites. Such supplemental feed or bait can be a useful management tool to attract animals to deliver oral vaccines and topical treatments or increase hunting and trapping success; nevertheless, it requires strict precautions against local environmental degradation, increased spread of infectious disease, and non-target animal use. Unfortunately, the recommendations for effective tick control using ‘4-Poster’ feeders are likely at odds with the effective management of environmental degradation and disease spread. In fact, the risks of intensifying the spread of disease may often outweigh the benefits gained from tick control. If treating deer with ‘4-Poster’ feeders is an absolutely necessary component in an IPM plan, we recommend monitoring feeding stations with camera traps to observe deer use, non-target species use, and social interactions at stations, and we suggest animal and environmental disease surveillance at or around feeders.

## Figures and Tables

**Table 1:** Deployment schedule for 4-Poster feeders and site and deer demographics for 3 study sites in Howard County, Maryland, 2017–2019. Density estimates were for 2019 and calculated by Howard County via FLIR helicopter counts.

Park	Size (ha)	Density Estimate (deer/km^2^)	# of ‘4-Poster’	1st Deployment	2nd Deployment	3rd Deployment
Blandair Regional Park	60.7	23.9	4	17 October 2017–27 December 2017	2 April 2018–10 January 2019	26 March 2019–26 August 2019
Cedar Lane Park	37.6	N/A	2	17 October 2017–27 December 2017	2 April 2018–10 January 2019	26 March 2019–9 December 2019
Rockburn Branch Park	168.0	16.6	9	18 October 2017–27 December 2017	11 April 2018–10 January 2019	26 March 2019–17 December 2019

**Table 2:** Model results of white-tailed deer occurrence distribution size for 95% and 50% contours comparing before and after feeders were refilled with corn (treatment) in Howard County, Maryland 2017–2019. Model effects include age of deer (age), specific park (park), sex of deer (sex), before or after feeder was filled (treatment), and GPS data collar or remotely downloaded data (data type).

Effect	95% Occurrence Distribution			50% Occurrence Distribution		
	χ2	df	*p*	χ2	df	*p*
Age	12.226	4	0.016	17.883	4	0.001
Park	26.351	2	0.000	27.233	2	0.000
Sex	27.713	1	0.000	28.482	1	0.000
Treatment	2.240	1	0.134	0.728	1	0.394
Data Type	NA	NA	NA	3.081	1	0.079

**Table 3:** Model results of distance from white-tailed deer occurrence distribution extent to all ‘4-Poster’ feeders for 95% and 50% contours in Howard County, Maryland 2017–2019. Model effects include age of deer (age), specific park (park), before or after feeder was filled (treatment), and GPS data collar or remotely downloaded data (data type).

Effect	95% Occurrence Distribution			50% Occurrence Distribution		
	χ2	df	*p*	χ2	df	*p*
Age	9.742	4	0.045	NA	NA	NA
Park	9.342	2	0.009	6.757	2	0.034
Treatment	0.084	1	0.772	2.051	1	0.152
Data Type	2.703	1	0.100	NA	NA	NA

**Table 4:** Model results of distance from white-tailed deer occurrence distribution extent to nearest ‘4-Poster’ feeder for 95 and 50% contours in Howard County, Maryland 2017–2019. Model effect includes before or after feeder was filled (treatment).

Effect	95% Occurrence Distribution			50% Occurrence Distribution		
	χ2	df	*p*	χ2	df	*p*
Treatment	0.735	1	0.391	4.50	1	0.034

**Table 5:** Model results testing white-tailed deer hourly location distances to nearest feeder before and after refilling (treatment) in Howard County, Maryland 2017–2019. Model effects include specific park (park), sex of deer (sex), before or after feeder was filled (treatment), and hour of day (hour).

Effect	*F*	df	*p*
Park	4.062	2	0.0172
Sex	6.057	1	0.0139
Treatment	5.048	1	0.0247
Hour	80739	8.739	<0.001

**Table 6:** Model results for two clustering analyses on white-tailed deer before and after ‘4-Poster’ feeder servicing in Howard County, Maryland 2017–2019. Model effects include before or after feeder was filled (treatment), hour of day (hour), and the number of deer eligible to be included for a specific hour (N).

Effect	Mean Nearest Neighbor			Average Pair-Wise Distance		
	Stat *(t, F)*	df	*p*	Stat *(t, F)*	df	*p*
Treatment	3.155	1	0.016	2.568	1	0.0103
Hour	4.750	3.909	<0.001	3.528	3.869	<0.001
N	430.645	4.638	<0.001	38.487	4.614	<0.001

**Table 7:** Model results for probability of deer visiting ‘4-Poster’ feeders in Howard County, Maryland 2017–2019. Model effects include sex of deer (sex), smooth tensor product of hour and day (TE(Hour,Day)), and smooth tensor product of hour and day by sex (TE(Hour,Day):sex).

Effect	*X* ^2^	Df	*p*
Sex	0.001	1	0.972
TE(Hour, Day)	176.9	36.591	<0.001
TE(Hour, Day):Sex	59	74.95	<0.001

## Data Availability

Data are available from the corresponding author upon request.
